# Rapid response to monotherapy with MEK inhibitor trametinib for a lung adenocarcinoma patient harboring primary *SDN1-BRAF* fusion: A case report and literature review

**DOI:** 10.3389/fonc.2022.945620

**Published:** 2022-08-19

**Authors:** Yang Yu, Min Yu, Yanying Li, Xiaojuan Zhou, Tian Tian, Yijia Du, Zegui Tu, Meijuan Huang

**Affiliations:** ^1^ Department of Thoracic Oncology and State Key Laboratory of Biotherapy, Cancer Center, West China Hospital, Sichuan University, Chengdu, China; ^2^ West China school of medicine, Sichuan University, Chengdu, China

**Keywords:** *BRAF* fusion, *SDN1-BRAF*, lung adenocarcinoma, trametinib, MAPK pathway

## Abstract

*BRAF* gene has been identified as an oncogenic driver and a potential target in various malignancies. *BRAF* fusions are one subtype of *BRAF* alterations with a rare frequency. Here, we first report a previously treated advanced lung adenocarcinoma patient with *de novo SND1-BRAF* fusion who achieves partial response to the MAK inhibitor trametinib. We also provide a literature review on targeted therapies for *BRAF* fusions.

## Introduction

*BRAF* gene encodes the RAF kinase and activates the MAPK pathway. It has emerged as an oncogenic driver and a potential therapeutic target in a wide variety of solid tumors (1, 2). Based on signaling mechanism, kinase activity, and sensitivity to inhibitors, *BRAF* mutations have been categorized into three functional classes: RAS-independent kinase-activating V600 monomers (class I), RAS-independent kinase-activating dimers (class II), and RAS-dependent kinase-inactivating heterodimers (class III) (3, 4). *BRAF* alterations occur in 4.4% of non-small cell lung cancer (NSCLC) patients (1). The most prevalent variant is the BRAF *V600* mutation, accounting for 1%–2% of BRAF-mutated NSCLC patients (5). However, *BRAF* fusions, one subtype of *BRAF* class II mutations, are only identified in 0.2% of NSCLC samples (1). The Food and Drug Administration (FDA) has approved dabrafenib plus trametinib to treat NSCLC patients with *BRAF V600* mutation (6). In contrast, *BRAF* fusions are not yet eligible for targeted therapies (4).

Unlike *BRAF* mutation, *BRAF* gene fusions activate the MAPK signaling pathway by inducing the removal of the auto-inhibitory N-terminal moiety (7). *BRAF* fusions exist in numerous solid tumors, including melanoma, glioma, thyroid cancer, pancreatic carcinoma, NSCLC, and colorectal cancer (1, 8). *BRAF* fusions can take on various forms. Ross et al. and Zehir et al. have reported 44 distinct types of *BRAF* fusions in multiple solid tumors, especially in NSCLC, such as *EPS15-BRAF*, *NUP214-BRAF*, *ARMC10-BRAF*, *BTF3L4-BRAF*, *AGK-BRAF*, *GHR-BRAF*, *ZC3HAV1-BRAF*, *TRIM24-BRAF*, *GK-BRAF*, *PJA2-BRAF*, *SND1-BRAF*, *MRPS33-BRAF*, and *PARP12-BRAF (* 1, 9). Unfortunately, reports on anti-*BRAF* therapies for NSCLC with *de novo BRAF* gene fusion are scarce.

The study shows a rapid response to trametinib monotherapy in the advanced lung adenocarcinoma patient with *de novo* SND1-BRAF fusion for the first time.

## Case report

A 60-year-old man was admitted to West China Hospital, Sichuan University in February 2017 with symptoms of expectoration and shortness of breath for 1 month. He was a current smoker (30 packs/years) with no family history of cancer. High concentration levels of serum carcinoembryonic antigen (CEA) (63.92 ng/ml) and neuron-specific enolase (NSE) (23.35 ng/ml) were revealed by blood tests. Right lung masses and solid nodules were visible on a chest computed tomography (CT) scan, which also revealed enlargement of the right hilar and mediastinal lymph nodes, focal thickening of the right pleura, and right pleural effusions. He underwent a CT-guided biopsy of the right lung mass, and the lesion was pathologically diagnosed as adenocarcinoma. Immunohistochemical examination of the right pleural effusions showed positivity of CK7, Napsin A, P63, and TTF-1 and negativity of CK5/6, ALK-V, and ROS-1, PD-L1 tumor proportion score (TPS) of 30% , which confirms that the metastatic adenocarcinoma originated from the lung. Next-generation sequencing (NGS) (Burning Rock, Guangzhou, China) with a panel of 295 cancer-related genes was conducted to examine the tumor tissue. The outcome was positive for *SND1-BRAF* (S10:B9) fusion (abundance 3.8%), whereas *EGFR*, *ALK*, *ROS1*, and other sensitive genes were all negative.

According to the Eighth Edition of the TNM Classification for Lung Cancer, the patient was classified as stage cT4N3M1a (cIVA) lung cancer. Therefore, the patient underwent four cycles of cisplatin/pemetrexed combined with bevacizumab as the first-line therapy. Then, the patient received maintenance treatment of pemetrexed/bevacizumab until the disease spread to his right thoracic cavity and left the adrenal gland. The disease progression was revealed in March 2020 through the chest and abdomen CT. The best response was partial response (PR) with a PFS of 36 months during first-line therapy. The major treatment-associated adverse events were Grade 1 gingival bleeding and Grade 2 leukopenia and neutropenia. Pembrolizumab (200 mg, Q3W) was administered to the patient as the second-line therapy. However, the patient’s right thoracic solid nodules continued to grow. Several treatment lines failed, including pembrolizumab (200 mg, Q3W) plus docetaxel (110 mg, Q3W) and pembrolizumab (200 mg, Q3W) plus anlotinib (10 mg D1–14, Q3W).

**Figure 1:**
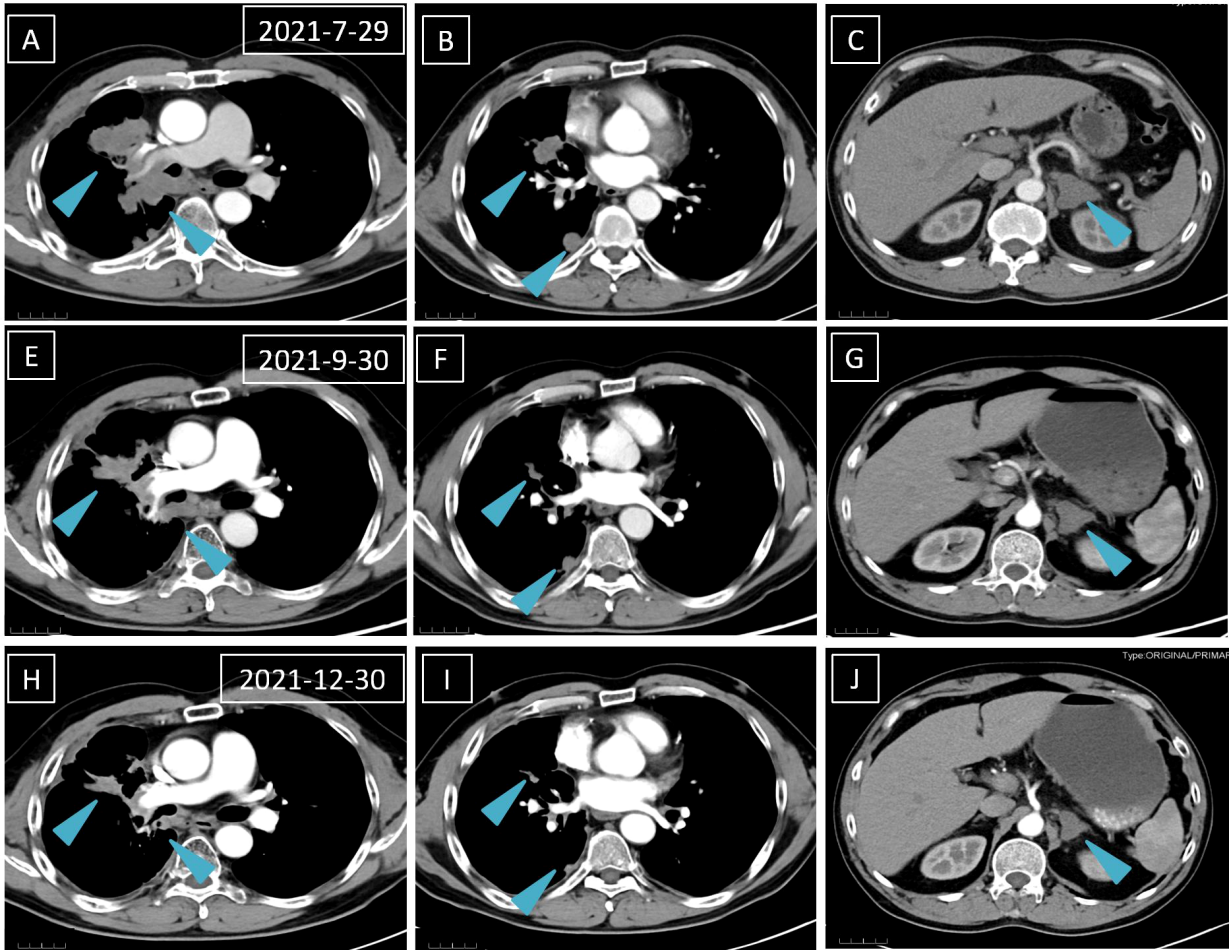
** **A computed tomography scan is performed before **(A–C)**, 1 month after **(D–F)**, and 4 months after **(H–J)** trametinib treatment. The blue arrows mean lung and left adrenal gland nodules.

**Figure 2:**
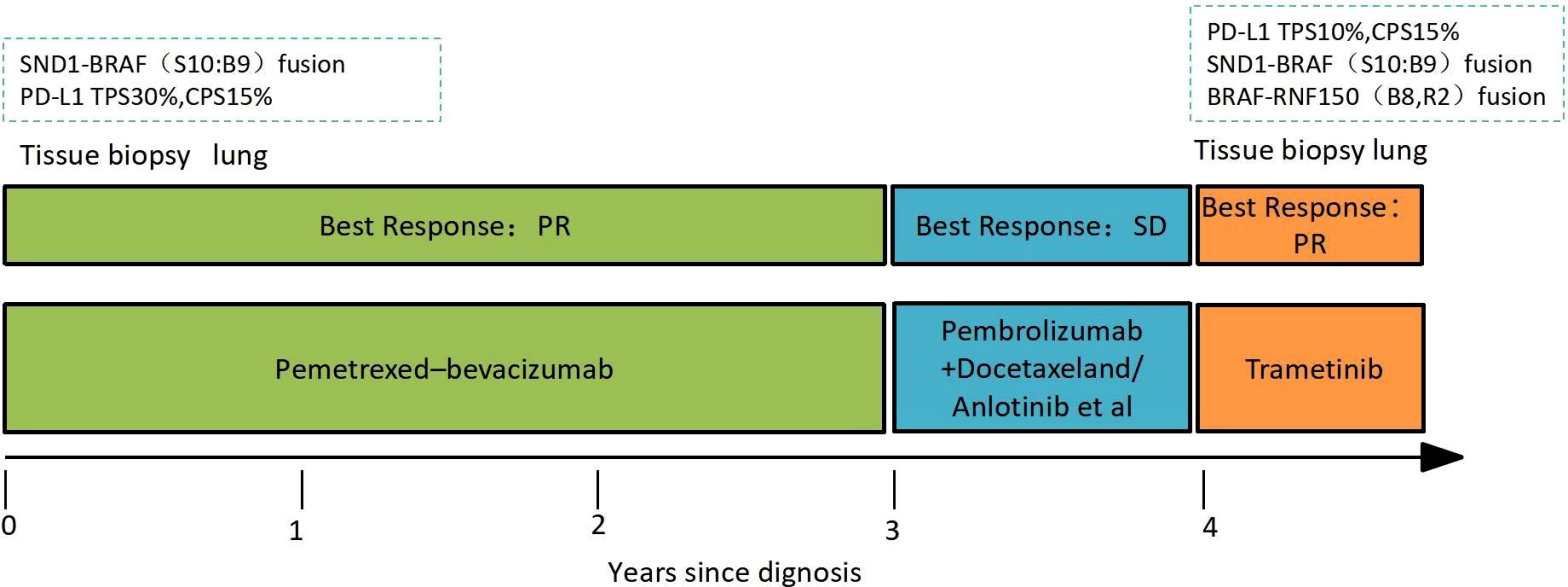
Timeline and duration of each regimen.

Laboratory results revealed an increased NSE level of 24.5 ng/ml in July 2021, and multiple lesions on the CT scan suggested disease progression. It should be noted that the NSE concentration remained normal throughout last year. Furthermore, a percutaneous right lung biopsy guided by CT confirmed the patient’s adenocarcinoma diagnosis. Whole-exome sequencing (WES) (Berry Oncology Corporation, Beijing, China) was conducted to analyze the biopsy specimen. The result showed the positivity of *SND1-BRAF* (S10:B9) and *BRAF-RNF150*(B8:R2)fusions with a PD-L1 TPS of 10% in August 2021. The patient received the MEK inhibitor trametinib (2 mg daily) from 1 September 2021. His cough was significantly relieved following 1-week trametinib treatment. The only adverse effect, which appeared 3 weeks after taking trametinib, was scattered acne with itching over the head and face (CTCAE 1 grade). After 4 months, the CT scan confirmed the patient with PR with a dramatic tumor shrinkage of 57% (**Figure 1**). He is now treated with trametinib. The overall timeline of diagnosis and treatment is displayed in **Figure 2**.

## Discussion

*BRAF* fusions occur at a rare frequency (1, 10). Moreover, there are no anti-*BRAF* treatment guidelines for lung cancer patients with *BRAF* fusions. The situation is further worsened by the dearth of case reports on anti-*BRAF* targeted therapy. Our patient is the first reported NSCLC case with *de novo SND1-BRAF* fusion responding to the MEK inhibitor trametinib.

**Figure 3:**
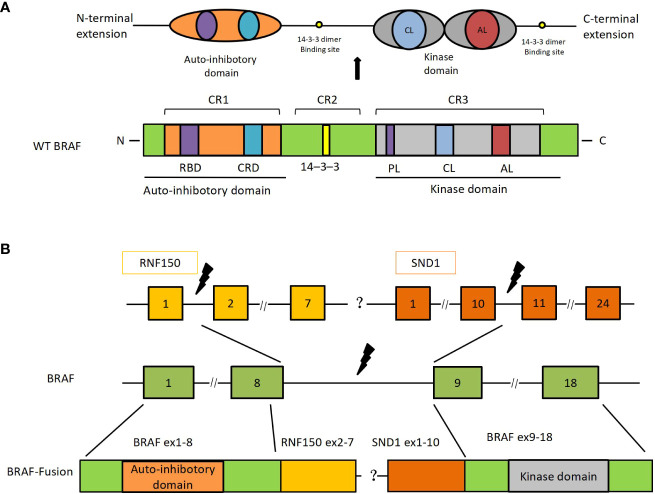
*SND1-BRAF* transcript fusion in metastatic lung adenocarcinoma. **(A)** The primary structure of *BRAF* and its functional domains. **(B)** *BRAF-RNF150* and *SND1-BRAF* fusions. RBD, ras-binding domain; CRD, cysteine-rich domain; PL, phosphate-binding loop; CL, catalytic loop; AL, activation loop; SND, staphylococcal nuclease domain; Ex, exon.

Despite the rarity of *BRAF* gene fusions, the alterations can be found in several solid tumors, including melanoma, glioma, thyroid cancer, pancreatic carcinoma, NSCLC, and colorectal cancer (1, 11). In contrast to *BRAF* monomer mutations, which often result in the mutation of the kinase domain sand, BRAF fusion proteins retain the normal kinase domain sand while inducing the loss of the auto-inhibitory N-terminal moiety (7) (**Figure 3**). Moreover, *BRAF* fusions exhibit variability due to their distinct partners, which have been documented in several papers (1, 9). However, there are no literature reports on *BRAF-RNF150*. Its role and mechanism in tumorigenesis and tumor development are still being investigated. *SND1-BRAF* fusion has been found in pancreatic cancer and lung adenocarcinoma. Nevertheless, no clinical investigation has shown any instances of *SND1-BRAF* fusion in solid malignancies. Preclinical research on pancreatic acinar cell carcinoma revealed that trametinib could significantly inhibit the growth of *SND1-BRAF* transformed cells. At the same time, TAK-632 exhibited a weak suppressive effect, and sorafenib showed no inhibitory effect (12). Hutchinson et al. studied *PAPSS1-BRAF* or *KIAA1549-BRAF* transfected 293H cells treated with vemurafenib or trametinib *in vitro* (13). The finding revealed that the mutant-specific *BRAF* inhibitor trametinib, rather than vemurafenib, might block the downstream signaling induced by the *PAPSS1-BRAF* or *KIAA1549-BRAF* fusion. Botton et al. found that six melanoma cell lines harboring *BRAF* fusions were resistant to first- and second-generation RAF inhibitors (14). By contrast, next-generation αC-IN/DFG-OUT RAF inhibitors blunted the activation of all cell lines, and showed synergistic effects when combined with the MEK inhibitors. Similarly, Usta et al. screened MAPK inhibitors using the *KIAA1549-BRAF* transfected glioma cell line (15) The result revealed that MEK inhibitors inhibited the MAPK signaling pathway with the lowest IC_50_s, followed by ERK and next-generation RAF inhibitors. A synergistic effect was observed in the combination of RAF and MEK inhibitors. In addition, Vojnic et al. identified four individuals with *EGFR*-mutated lung cancers that acquired BRAF rearrangements and showed secondary resistance to anti-*EGFR* therapy (16). The study further induced the *AGK-BRAF* fusion in H1975 (L858R+T790M), PC9 (ex19del), and HCC827 (ex19del) cell lines, and the cells also displayed osimertinib resistance. Furthermore, trametinib could synergistically suppress the proliferation of these cell lines when combined with osimertinib (16). Clinical references for our regimen included various studies describing anti-*BRAF* targeted therapies on different forms of *BRAF* fusions (**Table 1**). Pan-RAF inhibitors were an effective agent for targeting the *BRAF* fusions as demonstrated both in animal models and *in vitro*. Yao et al. reported that a dual RAF inhibitor BGB659 could inactivate RAF fusion proteins by blocking the ERK signaling pathway (22). Similarly, Peng et al. showed that LY3009120, a pan-RAF and RAF dimer inhibitor, could inhibit RAF isoforms (*ARAF*, *BRAF*, and *CRAF*) and occupy protomers in RAF dimers (23). Another *BRAF*-specific dimer breaker called PLX8394 was discovered by Yao et al. It showed a superior safety profile in clinical practice and preferentially suppressed the ERK signaling pathway in tumors driven by dimeric *BRAF* mutants while sparing RAF function in normal cells (24).

**Table 1:** Literature review on anti-*BRAF* therapies used in solid tumors with *BRAF* gene fusions.

Case	Histologic diagnosis	Age/Gender	Sample source	BRAF fusion	Therapy	PFS(month)	Response	OS(month)
Chew et al. (17)	Melanoma	40/F	Lung metastasis	SKAP2-BRAF	Trametinib	3 m	PR	4 m
Menzies et al. (18)	Melanoma	47/F	Brain metastasis	PPFIBP2-BRAF	Trametinib	NA	PR	8 m
	Melanoma	65/M	Subcutaneous calf metastasis	KIAA1549-BRAF	Trametinib	NA	SD (enlargement)	8 m
Subbiah et al. (19)	Spindle cell neoplasm	55/F	Primary tumor	KIAA1549-BRAF	Bevacizumab + temsirolimus + sorafenib	NA	SD (reduction)	NA
Isaacson et al. (20)	Papillary urothelial carcinoma	69/M	Liver metastasis	NRF1-BRAF	Trametinib	3 m	PR	NA
del Bufalo et al. (21)	Gangliogliomas	2/M	Primary tumor	KIAA1549-BRAF +BRAF V600E	Vemurafenib	NA	NA (reduction)	NA
Zhu YC et al. (10)	Lung cancer	60/M	Primary tumor	TRIM24-BRAF	Vemurafenib	3.5 m	PR	9 m
Wang CY et al. (14)	Lung cancer	66/M	Pleural metastasis	LIMD1-BRAF	Trametinib	7.4 m	PR	NA

M, male; F, female; PFS, progression-free survival; OS, overall survival; PR, partial response; SD, stable disease; NA, not available.

In conclusion, drugs targeting the MAPK pathway, particularly MEK inhibitors whose antitumor benefits have been shown in preclinical research and clinical case reports, may effectively treat *BRAF* fusion-related cancers (15). In addition, ERK and next-generation RAF inhibitors may have a synergistic antitumor impact across distinct classes of MAPK inhibitors. In this research, the NSCLC patient with primary *SDN1-BRAF* fusion receives continuous trametinib monotherapy, and it is encouraging to observe PR. Further research is required to understand the biochemical and oncogenic mechanisms and identify the targeted strategy in NSCLC patients harboring *BRAF* fusions.

## Data availability statement

The original contributions presented in the study are included in the article/supplementary material. Further inquiries can be directed to the corresponding author.

## Ethics statement

Written informed consent was obtained from the individual(s) for the publication of any potentially identifiable images or data included in this article.

## Author contributions

MH, Corresponding author, contributed to the conception of the study and helped perform the analysis with constructive discussions. YY contributed significantly to manage and treat these patients, analysis and wrote the manuscript. MY, YL, XZ, TT, YD, ZT helped manage and treat this patient. All authors contributed to the article and approved the submitted version.

## Acknowledgments

We would like to thank TopEdit (www.topeditsci.com) for English language editing of this manuscript.

## Conflict of interest

The authors declare that the research was conducted in the absence of any commercial or financial relationships that could be construed as a potential conflict of interest.

## Publisher’s note

All claims expressed in this article are solely those of the authors and do not necessarily represent those of their affiliated organizations, or those of the publisher, the editors and the reviewers. Any product that may be evaluated in this article, or claim that may be made by its manufacturer, is not guaranteed or endorsed by the publisher.
